# A Case Report of an Inflammatory Hepatocellular Adenoma Mimicking Hepatocellular Carcinoma in a Patient with Alcoholic Cirrhosis

**DOI:** 10.2174/0115734056447538260331115432

**Published:** 2026-04-03

**Authors:** Weon Jang, Woo Sung Moon, Ji Soo Song

**Affiliations:** 1 Department of Radiology, Jeonbuk National University Medical School, Research Institute of Clinical Medicine of Jeonbuk National University, Biomedical Research Institute of Jeonbuk National University Hospital, Jeonju, Korea;; 2 Department of Pathology, Jeonbuk National University Medical School, Research Institute of Clinical Medicine of Jeonbuk National University, Biomedical Research Institute of Jeonbuk National University Hospital, Jeonju, Korea

**Keywords:** Inflammatory hepatocellular adenoma, Hepatocellular carcinoma, Alcoholic liver cirrhosis, Serum amyloid A, C-reactive protein

## Abstract

**Background:**

Hepatocellular adenoma (HCA) is a benign liver tumor that can mimic hepatocellular carcinoma (HCC), especially in patients with cirrhosis. Inflammatory HCA, a subtype of HCA, is characterized by increased expression of inflammation-related proteins such as serum amyloid A (SAA). Distinguishing inflammatory HCA from other liver lesions, such as HCC, can be challenging on imaging, especially in cirrhotic livers.

**Case Presentation:**

A 44-year-old male with alcoholic liver cirrhosis was found to have a hepatic lesion in segment 8, which gradually increased in size from 15 mm to 43 mm over 47 months. The lesion exhibited arterial phase hyperenhancement and washout on delayed imaging. It fulfilled the Liver Imaging Reporting and Data System (LI-RADS) v2018 criteria for LR-5, indicating definite HCC. The patient underwent laparoscopic subsegmentectomy of segment 8 without prior biopsy. Histopathological examination confirmed the diagnosis of inflammatory HCA. Immunohistochemistry showed strong and diffuse expression of SAA and C-reactive protein, and no aberrant β-catenin expression. The lesion was initially diagnosed as HCC based on radiologic features; the final diagnosis was inflammatory HCA. The patient recovered well after surgery and was discharged nine days later.

**Conclusion:**

Inflammatory HCA can mimic HCC on imaging, especially in patients with alcoholic cirrhosis. In such cases, relying solely on imaging may lead to misdiagnosis. Correlation with clinical history, pathology, and immunohistochemical findings is important to avoid unnecessary surgery and ensure accurate diagnosis.

## 1 INTRODUCTION

Hepatocellular adenoma (HCA) is a benign neoplasm composed of mature hepatocytes. Microscopically, HCA exhibits minimal or no cytologic and architectural atypia and is genetically chromosomally stable, with relatively few mutations compared to hepatocellular carcinoma (HCC) [1]. HCA represents a heterogeneous entity that has been subclassified into several molecular and phenotypic subtypes: hepatocyte nuclear factor 1A (HNF1A)-inactivated HCA, β-catenin–activated HCA, inflammatory HCA, and unclassified HCA [2]. More recent studies have proposed expanding the classification into eight molecular subtypes based on gene expression profiles [3, 4]. Among these subtypes, inflammatory HCA is characterized by increased expression of inflammation-associated proteins such as serum amyloid A (SAA) and C-Reactive Protein (CRP) [5, 6].

A primary diagnostic concern is distinguishing inflammatory HCA from other hepatic nodules that share overlapping histological features, such as diffuse SAA expression, sinusoidal dilatation, and ductular reaction. Serum amyloid A-positive nodules represent a morphologic spectrum of hepatic lesions, including inflammatory HCA and Focal Nodular Hyperplasia-Like Nodules (FNH-LNs), characterized by diffuse SAA expression. While most SAA-positive nodules are inflammatory HCA, FNH-LNs may closely resemble inflammatory HCA in alcoholic cirrhosis both radiologically and histologically [7, 8].

Differentiating SAA-positive nodules from HCC can be particularly challenging. On gadoxetate-enhanced MRI, they may demonstrate arterial phase hyperenhancement (APHE), washout in the delayed phase, and hypointensity on the hepatobiliary phase, which are typically seen in HCC [9]. In addition, a previous study reported that approximately 67% of SAA-positive nodules show hepatobiliary phase hypointensity [8]. Therefore, in cirrhotic patients with alcohol-related liver disease, SAA-positive nodules may be misinterpreted as HCC.

Herein, we report a case of inflammatory HCA in a 44-year-old male with alcoholic liver cirrhosis, initially suspected to be HCC based on clinical and imaging features. We focus on imaging pitfalls and the importance of histopathological correlation.

## 2 CASE REPORT

**Fig. (1):**
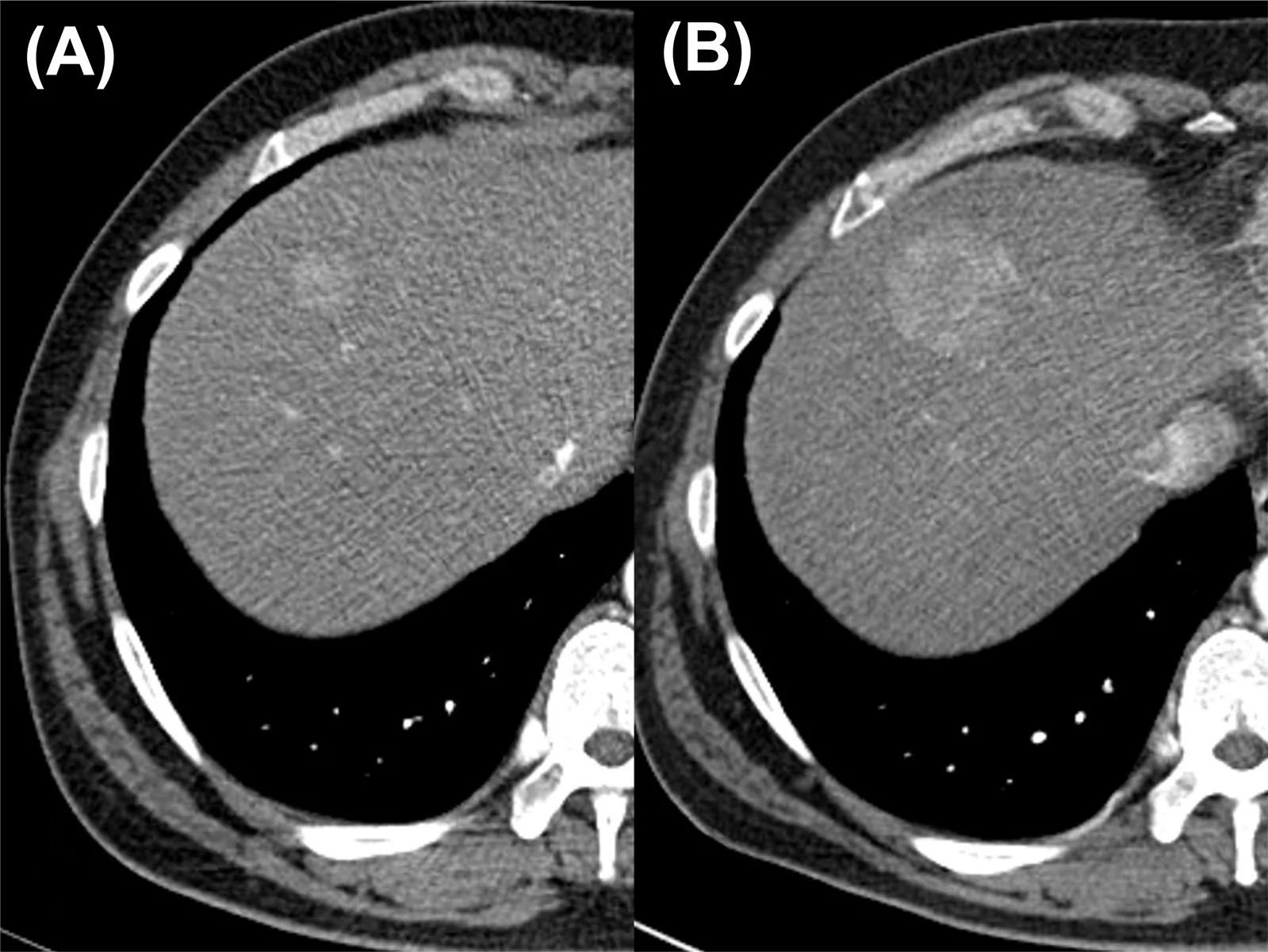
(**A**) Arterial phase CT image obtained 4 years prior shows a 1.5 cm hypervascular mass in segment 8 of the liver. (**B**) Follow-up arterial-phase CT image demonstrates interval growth of the hypervascular mass to 4.3 cm.

A 44-year-old male with a history of chronic alcohol consumption, with an average daily ethanol intake of approximately 100 g for over 20 years, was diagnosed with alcoholic liver cirrhosis. A hepatic lesion was identified, which increased in size from 15 mm to 43 mm over 47 months (Fig. **1A**, **B**). Serologic tests for hepatitis B and hepatitis C were negative. Tumor markers were as follows: alpha-fetoprotein, 15.5 ng/mL (normal ≤ 8.1); CA 19-9, 100.71 U/mL (normal ≤ 37.0); and protein induced by vitamin K absence (PIVKA-II), 48 mAU/mL (normal ≤ 40). Liver function was preserved, corresponding to a Child–Pugh score of 6, class A.

**Fig. (2):**
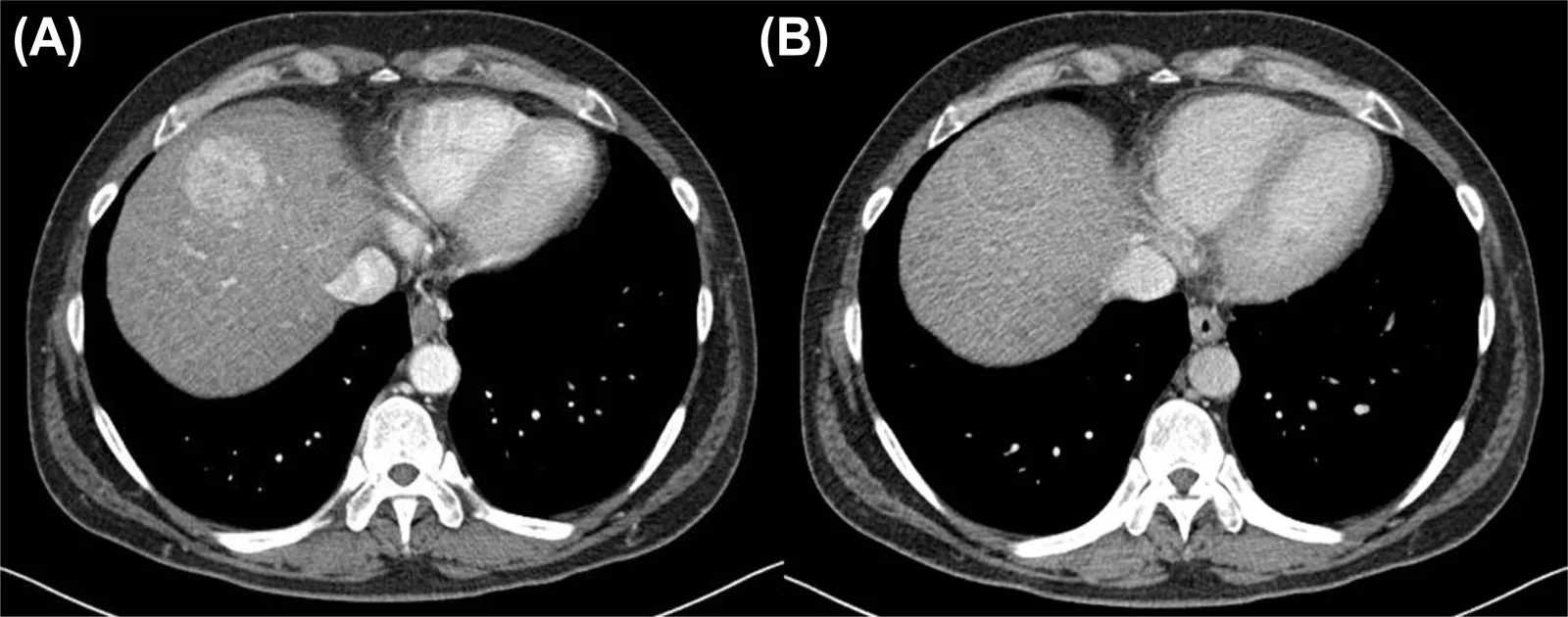
(**A**) The arterial phase of contrast-enhanced CT shows marked hyperenhancement of the hepatic lesion. (**B**) The delayed phase image demonstrates a washout appearance of the lesion.

**Fig. (3):**
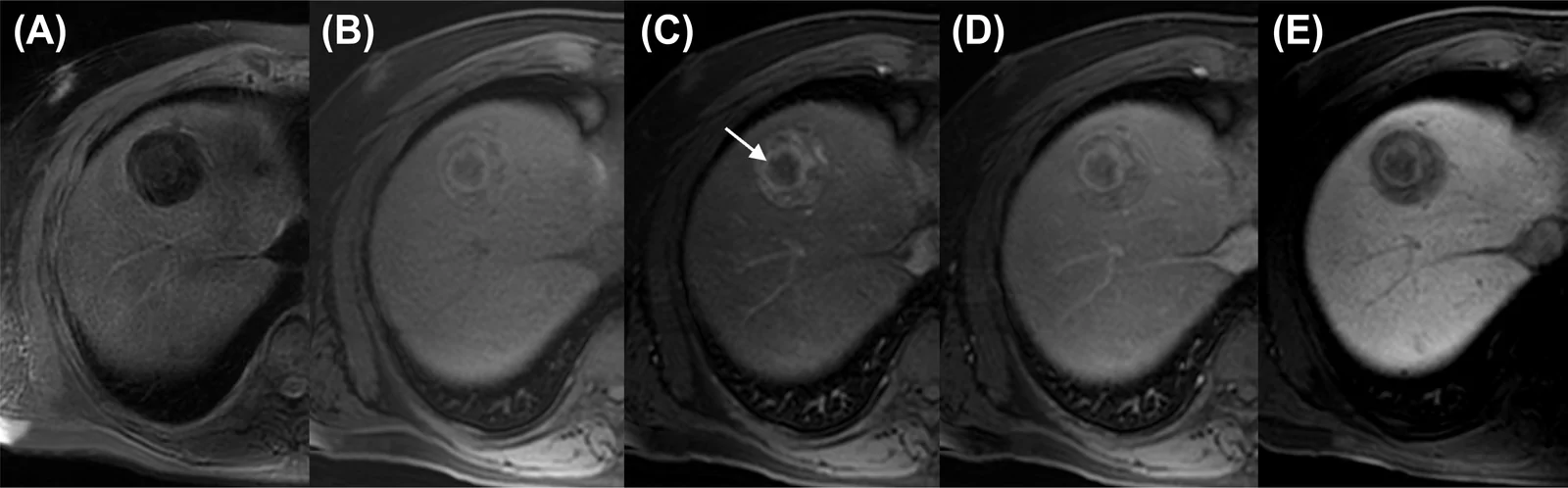
In gadoxetic acid-enhanced MRI of the liver, (**A**) the T2-weighted image shows heterogeneous low signal intensity of the lesion, while (**B**) the T1-weighted image reveals heterogeneous high signal intensity. (**C**) In the arterial phase, the lesion demonstrates strong enhancement with a nodule-in-nodule appearance (arrow), followed by (**D**) washout appearance on the portal venous phase. (**E**) The hepatobiliary phase image shows hypointensity of the lesion.

On contrast-enhanced abdominal CT, the round-shaped hepatic mass showed arterial enhancement and delayed washout (Fig. **2A**, **B**). MRI revealed a heterogeneous mass with T2 hypointensity and T1 hyperintensity (Fig. **3A**, **B**). A nodule-in-nodule appearance was observed. The lesion demonstrated heterogeneous arterial enhancement and washout on the portal venous phase, and hypointensity on hepatobiliary phase imaging (Fig. **3C**-**E**). Given the imaging findings and the patient’s cirrhotic background, the lesion met the Liver Imaging Reporting and Data System (LI-RADS) v2018 criteria for LR-5, indicating definite hepatocellular carcinoma; therefore, surgical resection was pursued without prior biopsy.

**Fig. (4):**
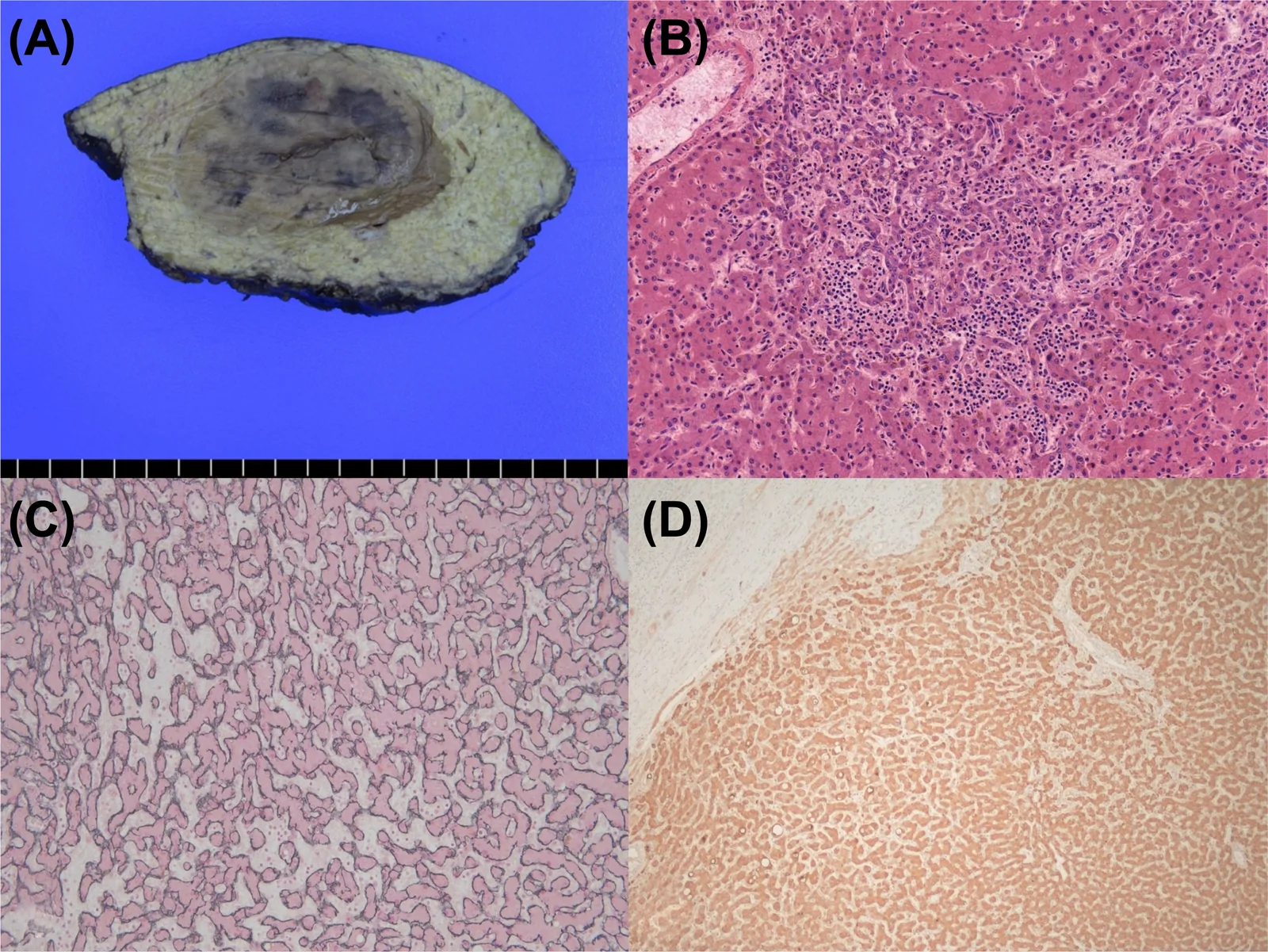
(**A**) The resected tumor is well-demarcated and displays internal hemorrhage in a cirrhotic background. (**B**) Histologically, the tumor is composed of benign-appearing hepatocytes arranged in thin trabeculae, with an inflammatory infiltrate, ductular reaction, and thin-walled arteries. (**C**) Reticulin stain highlights sinusoidal dilatation. (**D**) Immunostaining for serum amyloid A reveals diffuse, strong positivity in tumor cells.

The patient underwent a laparoscopic subsegmentectomy of segment 8. Intraoperative gross findings revealed a cirrhotic liver with surface nodularity. Intraoperative blood loss was minimal, and no blood transfusion was required. The postoperative course was uneventful, and the patient was discharged nine days after surgery. Histopathological examination revealed features consistent with inflammatory HCA (Figure **4**). Immunohistochemistry showed strong, diffuse expression of SAA and CRP. No aberrant nuclear or cytoplasmic β-catenin expression was observed.

## 3 DISCUSSION

The final diagnosis was inflammatory HCA arising in a cirrhotic liver, mimicking HCC both radiologically and clinically. While HCC commonly develops in the setting of chronic liver disease or cirrhosis, HCA has historically been considered a lesion of non-cirrhotic livers. However, emerging evidence suggests that HCA, particularly the inflammatory subtype, may occur in patients with underlying liver diseases, including non-alcoholic steatohepatitis and alcoholic liver disease [8, 10, 11]. This case indicates that inflammatory HCA can arise in cirrhotic livers and may closely mimic HCC based on imaging and clinical features. In particular, SAA-positive nodules may show imaging features similar to HCC, which can cause a significant diagnostic challenge. To our knowledge, only a limited number of cases of inflammatory HCA arising in cirrhotic livers have been reported, underscoring the exceptional nature of this case. Recognizing such cases is essential for appropriate diagnosis and management.

The major risk factors for inflammatory HCA include estrogen exposure from long-term oral contraceptives, aromatase-mediated estrogen production in adipose tissue, which is commonly associated with obesity or alcohol consumption, and underlying metabolic liver disease [4, 11]. The patient’s chronic alcohol consumption likely contributed to increased estrogen exposure, which may have contributed to the development of HCA. In addition, the presence of cirrhosis highly suggested HCC during the initial imaging evaluation. However, serologic tests excluded hepatitis B and C, and cirrhosis was considered alcohol-induced rather than due to chronic viral hepatitis. This clinical information could be another diagnostic clue for inflammatory HCA.

The Liver Imaging Reporting and Data System (LI-RADS) provides a structured framework for categorizing liver observations based on imaging features in patients at high risk for HCC, including those with cirrhosis due to alcohol [12]. According to LI-RADS, lesions ≥10 mm that show arterial-phase hyperenhancement and washout on delayed imaging are classified as LR-5, indicating a definitive diagnosis of HCC [13]. This structured system enhances diagnostic consistency and confidence in typical clinical settings.

Although LI-RADS provides a reliable framework for HCC diagnosis in at-risk populations, it does not incorporate histopathologic or molecular correlations, which limits its specificity in rare mimickers such as HCA. In this case, the hepatic lesion satisfied the major criteria for LR-5. Based on imaging, the lesion would have been categorized as HCC. This indicates that relying on LI-RADS categorization alone has diagnostic limitations in atypical clinical settings. Although alcoholic cirrhosis places the patient within the LI-RADS-eligible population, rare benign mimickers such as inflammatory HCA can still occur and fulfill imaging criteria for LR-5. Therefore, careful correlation with immunohistochemical and clinical data is essential to avoid unnecessary interventions or misdiagnosis.

Pathologically, the differential diagnosis of inflammatory HCA includes focal nodular hyperplasia-like nodules and well-differentiated HCC, particularly in alcoholic cirrhosis. Inflammatory HCA typically shows diffuse expression of inflammatory markers such as serum amyloid A and C-reactive protein, whereas focal nodular hyperplasia-like nodules generally demonstrate preserved reticulin framework and characteristic ductular reactions. Previous studies have shown that SAA–positive hepatocellular nodules arising in alcoholic cirrhosis share clinicopathological features with inflammatory HCA and may closely mimic HCC. In the present case, the pathological findings were consistent with inflammatory HCA [8].

Typical imaging features of inflammatory HCA include T2 hyperintensity, the “atoll sign,” arterial phase hyperenhancement (APHE), and persistent delayed enhancement [6, 14, 15]. In contrast, the present case showed T2 hypointensity, which was not consistent with either inflammatory HCA or HCC. According to the pathologic report, this T2 hypointensity was likely associated with internal hemorrhage within the mass. This atypical hemorrhagic feature contributed to the diagnostic challenge. Rather, APHE, delayed washout, and HBP hypointensity mimicked HCC. This case emphasizes the diagnostic dilemma of distinguishing inflammatory HCA from HCC in a cirrhotic liver, especially when imaging findings are atypical.

## CONCLUSION

We report a rare case of inflammatory HCA arising in a cirrhotic liver that radiologically mimics HCC and fulfills LI-RADS LR-5 criteria. Although surgical resection was appropriate in this case due to the high suspicion of malignancy based on clinical and imaging findings, this case underscores the diagnostic challenge posed by rare benign mimickers such as inflammatory HCA in cirrhotic patients.

In select cases with atypical imaging features or uncertain clinical context, considering inflammatory HCA in the differential diagnosis may lead to additional diagnostic workup, such as biopsy, and help avoid unnecessary surgical interventions. Accurate diagnosis requires correlation of imaging findings with clinical and histopathologic information. Radiologists and clinicians should be aware of rare but important benign mimickers to optimize patient management and reduce the risk of overtreatment.

## Data Availability

The data and supportive information are available within the article.

## LIST OF ABBREVIATIONS

HCA: Hepatocellular Adenoma
HCC: Hepatocellular Carcinoma
SAA: Serum Amyloid A
CRP: C-Reactive Protein
APHE: Arterial Phase Hyperenhancement
LI-RADS: Liver Imaging Reporting and Data System
FNH-LNs: Focal Nodular Hyperplasia-Like Nodules

## STANDARDS OF REPORTING

CARE guidelines were followed.
